# Supplementary material for: Causal Relationship between Aging and Anorexia Nervosa: A White-Matter-Microstructure-Mediated Mendelian Randomization Analysis
Source: Biomedicines. 2024 Aug 16;12(8):1874. doi: 10.3390/biomedicines12081874 (PMC11351342; doi:10.3390/biomedicines12081874)
Supplement: Supplementary file 1 [file biomedicines-12-01874-s001.zip › Supplementary Figure/Figure S8/Leaveoneout plot of MR analysis for TL to ubm-b-2070.pdf]

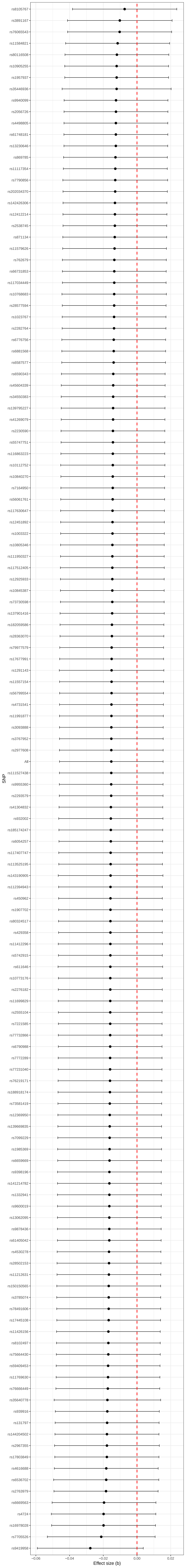
rs8105767
rs3891167
rs76065543
rs11584821
rs80116508
rs10905255
rs1957937
rs35446936
rs9940099
rs2056726
rs4498805
rs61748181
rs13230646
rs869785
rs11117354
rs7790856
rs202034370
rs142426306
rs12412214
rs2538745
rs871134
rs11579626
rs762679
rs66731853
rs117034449
rs10768683
rs28577594
rs1023767
rs2282764
rs6776756
rs6881568
rs6587577
rs6590343
rs45604339
rs34550383
rs139795227
rs41269079
rs2230590
rs55747751
rs116863223
rs10112752
rs10840270
rs7164950
rs56061761
rs117630647
rs12451892
rs1003322
rs10805346
rs111950327
rs117512405
rs12925933
rs10845387
rs73730598
rs137901416
rs182059586
rs28363070
rs79977579
rs17677991
rs1291143
rs11557154
rs56799554
rs4731541
rs11991877
rs3093888
rs3767952
rs2977608
All
rs111527438
rs9955360
rs2293579
rs41304832
rs932002
rs185174247
rs6054257
rs117407747
rs113525195
rs143190905
rs112394943
rs450962
rs1907702
rs80324517
rs429358
rs11412296
rs5742915
rs611646
rs10773176
rs2276182
rs11699829
rs2555104
rs7221585
rs77732866
rs6790988
rs7772289
rs77231040
rs76219171
rs188918174
rs73581419
rs12369950
rs139669835
rs7099229
rs1985369
rs6659669
rs9398196
rs141214782
rs1332941
rs9600019
rs13062095
rs9878436
rs61405042
rs4530278
rs28502153
rs11212631
rs150150565
rs3785074
rs78491606
rs17445108
rs11426156
rs8102497
rs75664430
rs59409453
rs11769630
rs76666449
rs35640778
rs939916
rs131797
rs144204502
rs2967355
rs17803849
rs4616688
rs6536702
rs2763979
rs6669563
rs4724
rs16978028
rs7705526
rs9419958
SNP
−0.06
−0.04
−0.02
0.00
0.02
Effect size (b)
